# Supplementary material for: Sorption of Bisphenol A as Model for Sorption Ability of Organoclays
Source: Molecules. 2022 Jul 6;27(14):4343. doi: 10.3390/molecules27144343 (PMC9316034; doi:10.3390/molecules27144343)
Supplement: Supplementary file 1 [file molecules-27-04343-s001.zip › molecules-1774909-supplementary.pdf]

molecules

Supplementary Materials

Sorption of bisphenol A as model for sorption ability of organoclays

Issaka Garikoé[1], Boubié Guel[1,*] and Ingmar Persson[2]

Introduction section

It was shown that the sorption of BPA remained fairly constant in the *pH* range 2-8 [8,11]. BPA has two acidity constants, pK_{a1} = 9.6 and pK_{a2} = 11.2 [18] and its acid-base distribution is presented in **Figure S1**. BPA (H_2A) is a neutral molecule as long as pH is below pK_{a1}. When $pK_{a1} \leq pH \leq pK_{a2}$, one of the phenol groups becomes deprotonated, **Figure S1**. BPA starts to deprotonate around pH = 8 (pK_{a1} = 9.6) and to divalent anions at around pH = 10 (pK_{a2} = 11.2).

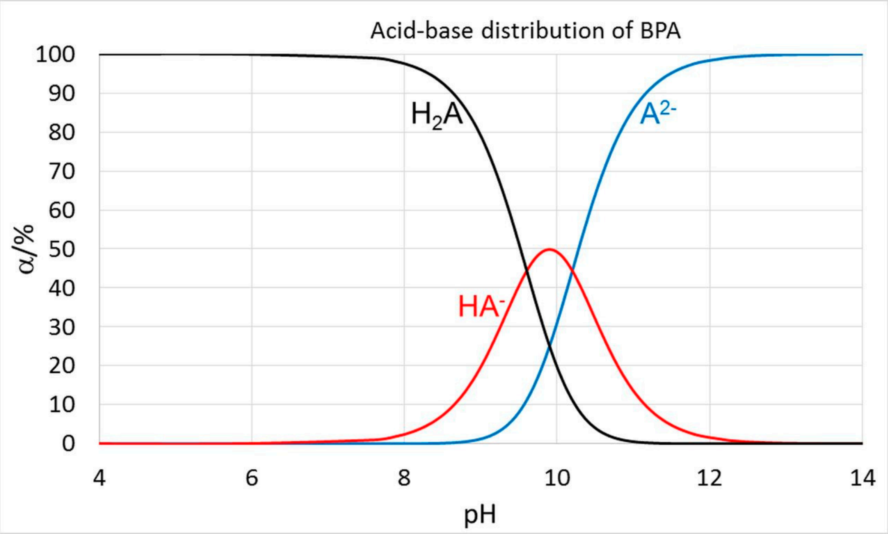

Figure S1. Acid-base distribution of BPA, neutral form: AH_2; BPA monoionized form: AH^- and BPA bionized form: A^{2-}.

2. Materials and Experimental Methods section

2.1. Materials

2.2. Experimental Methods

Organosmectites synthesis

The synthesis of organoclays was undertaken by the following procedure: 4 g of dried clay (powder size, ≤80 µm) and a well-defined amount of surfactant, which represents a level of 0.5 CEC, 1.0 CEC, 1.5 CEC and 2.0 CEC of the used clay, was placed in an agate mortar. The mixture was ground for 10-15 min to get a homogeneous powder. The treated clays were washed with distilled water to get rid of the bromide ions as checked by the addition of an aqueous solution of silver nitrate, dried at room temperature, and further dried in oven at 100 °C for 4 hours. The dried modified clays were ground in an agate mortar and stored in closed bottles. The modified clays were labelled as surfactant loading-type of surfactant-original location of the clay, e.g. 0.5 CEC-C_{12}-AH.

Adsorption measurements

5000 mg of BPA were dissolved in 1 L of ethanol because of its low solubility in water. All further solutions of BPA were prepared by diluting a well-defined volume of the stock solution in distilled water.

To determine the appropriate adsorbent dose for an efficient removal, different masses varying from 0.1 to 1.0 g of the raw clays or their organoclays and 40 mL of BPA solution at 100 mg/L were placed in a bottle of 60 mL. The bottles containing the mixture were then shaken with rotary or horizontally batch shaker during 12 hours at the ambient temperature.

The effect of the agitation time on sorption was carried out by placing 200 mg of the adsorbent (clays and organoclays) and 40 mL of a BPA solution at 100 mg/L in a bottle of 60 mL. These were shaken using a horizontally batch shaker for various agitation times from 10 to 120 minutes at the ambient temperature. The amount of BPA adsorbed for each interval of time was calculated and the obtained results allowed the determination of the minimal agitation time required to reach the equilibrium and the evaluation of the pseudo-first and pseudo-second kinetics models.

The amount of the adsorbed BPA in all of experiments was calculated using the following equation:

$$Q_e = [(C_i\text{-}C_e) \times V]/M \times 1000 \quad (1)$$

where: Q_e is the quantity of BPA adsorbed on the adsorbent (mg/g), C_i and C_e are initial and equilibrium concentrations of BPA (mg/L) respectively, V is the volume (mL), and M is the adsorbent mass (g).

The percentage of BPA removed is calculated using the equation 2.

$$\%\ \text{BPA} = [(C_i\text{-}C_e) \times 100]/C_i \quad (2)$$

where: % BPA is the percentage of BPA removed, C_i and C_e initial and equilibrium concentrations of BPA (mg/L).

The pseudo-first mode (Ho and McKay 1999) is used to determine the capacity of the adsorbents to uptake the adsorbate in a liquid/solid phase. The equations corresponding to these models are expressed below:

$$\mathrm{d}q_t/\mathrm{d}t = k_1 \cdot (Q_{e1} - Q_t) \quad (3)$$

where: k_1 is the rate constant of pseudo-first order adsorption (min^{-1}), Q_{e1} is the quantity of BPA adsorbed at equilibrium (mg/g), Q_t is the quantity of BPA adsorbed at any time t (mg/g).

The integration of the above equation, from t = 0 to t = t and q_t = 0 to q_t = Q_t as boundaries, gives the equation 4:

$$\ln(Q_e - Q_t) = \ln Q_{e1} - k_1 \times t \quad (4)$$

The constants are determined by plotting ln (Q_e-Q_t) versus t.

The pseudo-second order equation considered that the adsorption capacity is proportional to the number of active sites available on the adsorbent surface and its equation is written below:

$$\mathrm{d}q_t/\mathrm{d}t = k_2 \cdot (Q_{e1} - Q_t)^2 \quad (5)$$

where: k_2 is the rate constant for pseudo-second order adsorption (g/mg·min), Q_{e2} = amount of BPA adsorbed at equilibrium (mg/g), Q_t = amount of BPA adsorbed at any time t (mg/g).

The integration of equation 5, for the boundaries from t = 0 to t = t and q_t = 0 to q_t = Q_t, leads to equation 6:

$$t/Q_t = 1/[k_2 \times (Q_{e2})^2] + t/Q_{e2}) \quad (6)$$

The constants can be determined from the linear plot of t/Q_t against t.

Weber and Morris (Weber and Morris, 1963) suggested the use of intraparticle diffusion model to describe the rate determining step of the adsorption because they found the adsorbate removal to be proportional to $t^{1/2}$ rather than t and that the pseudo-first and pseudo-second order models are limited to furnish the diffusion mechanism. The following equation was employed to evaluate the intraparticle diffusion model.

$$Q_t = K_{diff} \times t^{1/2} + \text{constant} \quad (7)$$

where, K_{diff} is the constant of intraparticle diffusion (mg/g.min$^{1/2}$).

K_{diff} is determined by plotting Q_t versus $t^{1/2}$. When the obtained curve is a straight line, the intraparticle diffusion intervened in the process. If this line passes through the origin, the intraparticle diffusion is considered to be the controlling step of the adsorption.

The ratio of adsorbate (BPA) concentration to adsorbent (clays and organoclays) was studied by adding 40 mL of BPA solution with a concentration varying from 25 to 1000 mg/L for 200 mg of organoclays which were already placed in a bottle of 60 mL and the whole was shaken with a horizontally batch shaker during 12 hours at the ambient temperature. The measured pH values of the prepared BPA solutions were ranged from 5 to 6.

The Langmuir and Freundlich classic isotherms were applied and discussed in the present study in order to understand the adsorption process. Langmuir isotherm describes the interaction between the adsorbate and the adsorbent surface. It admits that a monolayer adsorption of adsorbate is realized on a homogeneous adsorbent. The adsorbent's specific sites adsorb and no further adsorption is realized at these sites. Thus, the driving force and surface area are strongly involved in the adsorbate removal on the adsorbent surface (Langmuir 1918). The Langmuir equation is expressed as below:

$$Q_e = x/M = K_L \times C_e / (1 + b_L \times C_e) \quad (8)$$

where: Q_e (mg/g) is the maximum amount of the adsorbate per unit of masse of adsorbent, K_L (L/mg) is the Langmuir constant related to the affinity of the binding sites.

The Langmuir isotherm can be expressed by a separation factor, R_L, defined by Weber and Chakkravorti (Weber and Chakkravorti 1974) such as:

$$R_L = 1/ (1 + K_L \times C_0) \quad (9)$$

The isotherm is classified as unfavorable for $R_L > 1$, favorable for $0 < R_L < 1$, linear for $R_L = 1$, or irreversible for $R_L = 0$.

The Freundlich isotherm is an empirical equation based on sorption of adsorbate on heterogeneous surfaces with sites having various affinities. The sorption starts with sites having stronger fixation and decreases progressively with increasing degree of site occupation (Freundlich 1906). The Freundlich isotherm is expressed as:

$$Q_e = K_F \cdot C_e^{1/n} \quad (10)$$

where: K_F (mg/g) is the Freundlich constants related to sorption capacity, C_e (mg/L) is the equilibrium concentration and n is the sorption intensity of the adsorbent.

K_F defines the amount of BPA adsorbed onto the adsorbent per unit mass at the equilibrium. When the value of $1/n < 1$, the adsorption corresponds to a normal Langmuir isotherm while $1/n > 1$ indicates cooperative adsorption (Fytianos and Kokkalis 2000).

The Temperature effect on the adsorption was carried out using 40 mL of BPA solution at 100 mg/L and 200 mg of adsorbent (organoclays). These were placed in a beaker and the mixture was agitated with a heating-agitator at 303, 313, and 323 K temperatures for 4 hours. The amount of BPA adsorbed at each temperature was determined. The effect of the temperature was used to evaluate the thermodynamic parameters during adsorption process. The thermodynamic constants such as Gibbs free energy (ΔG), enthalpies change (ΔH), and entropy changes (ΔS) were estimated using the equations presented below. The distribution constant of the adsorption K_d was calculated as follows (Park et al. 2014):

$$K_d = [(C_0\text{-}C_e)/C_e]\ V/m = Q_e/C_e \quad (11)$$

where V is the concentration of the suspension (mg/L), m is the mass of the adsorbent (g), C_0 and C_e represent the initial and equilibrium concentrations of BPA (mg/L) respectively.

The equilibrium adsorption constant K^o is obtained from the plot between $\ln K_d$ and C_e and the intercept of the plot gives the value of ln K^o which enables to calculate the free energy change (ΔG).

$$\Delta G = -RT\cdot\ln K^\circ \quad (12)$$

where R is a universal constant (8.314 J/mol·K) and T is the temperature (K).

$$\Delta G = \Delta H - T\Delta S \quad (13)$$

The enthalpy and entropy changes, ΔH and ΔS can be calculated from the following equation:

$$\ln K^\circ = -\Delta H/RT + \Delta S/R \quad (14)$$

The plot of $\ln K^o$ versus $1/T$ is linear and the slope and the intercept of the plot are -$\Delta H/R$ and $\Delta S/R$, respectively.

The *pH* effect on the adsorption of BPA was done by adjusting the *pH* of a solution of 100 mg/L from *pH* 2 to 12 and further 40 mL of each prepared solution and 200 mg of organoclays was placed in a bottle of 60 mL and the whole has been shaken for 12 hours with a horizontally shaker. The amount of adsorbed BPA at each *pH* was determined for every organoclay by HPLC measurements as described below.

After agitation on all case, the suspension was filtered through 0.45 µm nylon acrodisc or hydrophilic PTFE membrane filters. High-performance liquid chromatography (HPLC, Agilent HP 1100) system coupled with a Luna 5 µl C_{18} column (Phenomenex Pty Ltd.) and a DAD (UV-visible) detector was used to determine the concentration of BPA in the supernatants. The mobile phase was a mixture of acetonitrile and deionized water (1V/1V) and the detection wavelength was 278 nm. A flow rate of 1 mL/min and an injection volume of 20 µL were employed for the analyses.

3. Results and Discussion section

3.1. Characterization of BPA-clays and BPA-organoclays

3.1.1. X-ray diffraction

Table S1. Basal spacing, d_{001}/Å, of organoclays before and after BPA sorption.

Organoclays	d_{001}(Å) before BPA sorption	d_{001}(Å) after BPA sorption
1.0 CEC-C_{12}-AH	18.0	18.0; 27.8
1.0 CEC-C_{12}-DI	16.5	27.8
2.0 CEC-C_{12}-DI	19.7; 24.5	38.1
1.0 CEC-C_{14}-AH	21.8	32.1; ≥ 38.1
1.0 CEC-C_{14}-DI	24.5	32.1
1.0 CEC-C_{16}-DI	27.8	27.8; ≥ 38.1
2.0 CEC-C_{16}-AH	19.7; ≥ 38.1	≥ 38.1
1.0 CEC-2C_{12}-DI	16.5; 32.1	18.0; ≥ 38.1
2.0 CEC-2C_{12}-AH	18.0; ≥ 38.1	18.0; ≥ 38.1

3.1.2. Infrared spectroscopy

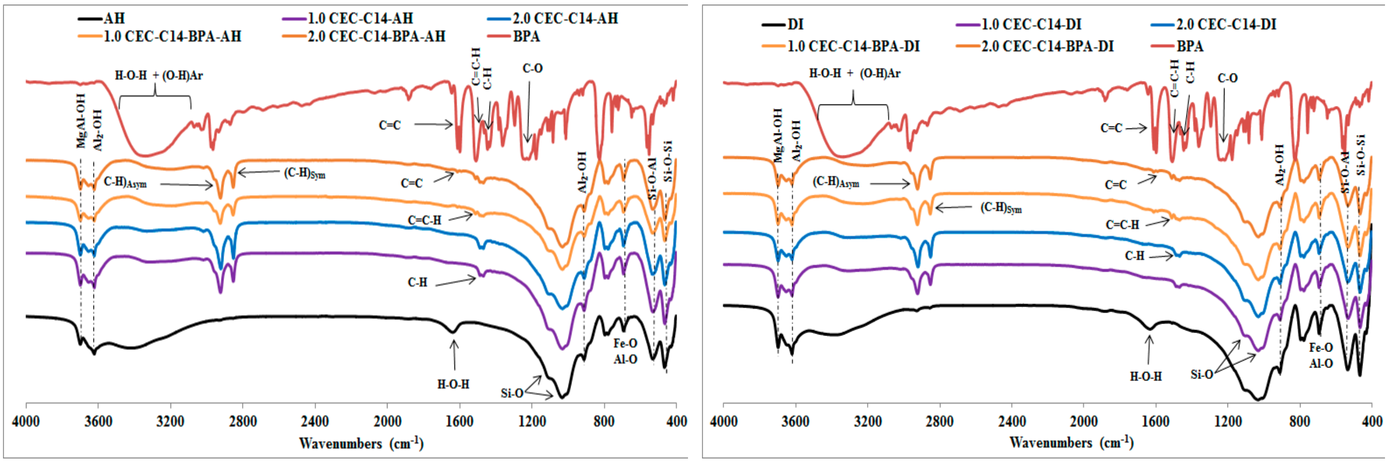

(**a**) (**b**)

Figure S2. IR spectra of (**a**) AH, BPA, C_{14}-AH, C_{14}-BPA-AH, (**b**) DI, BPA, C_{14}-DI, C_{14}-BPA-DI.

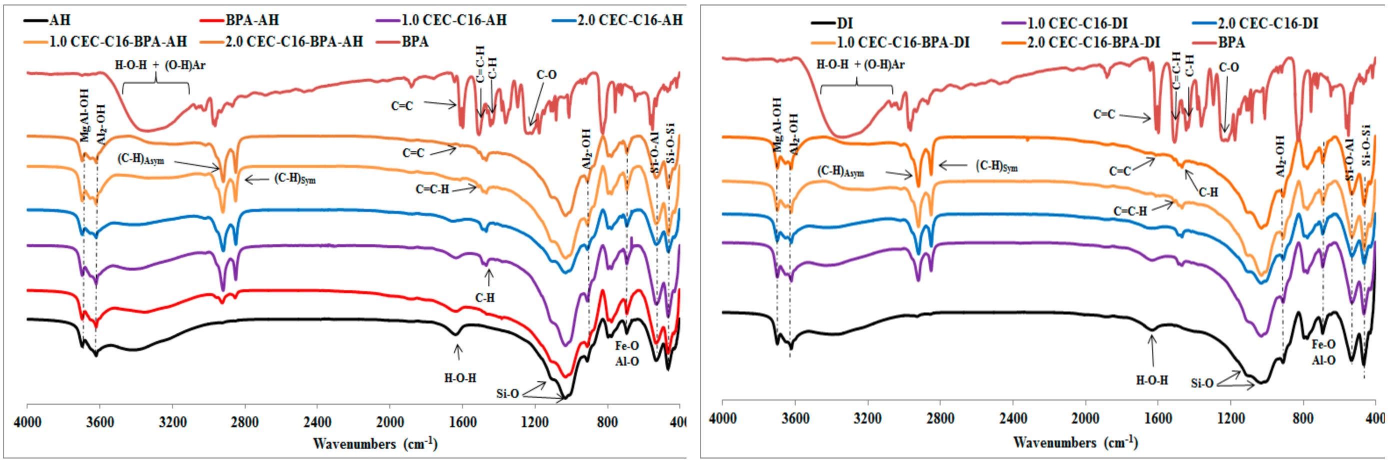

(**a**) (**b**)

Figure S3. IR spectra of (**a**) AH, BPA, C_{16}-AH, C_{16}-BPA-AH, (**b**) DI, BPA, C_{16}-DI, C_{16}-BPA-DI.

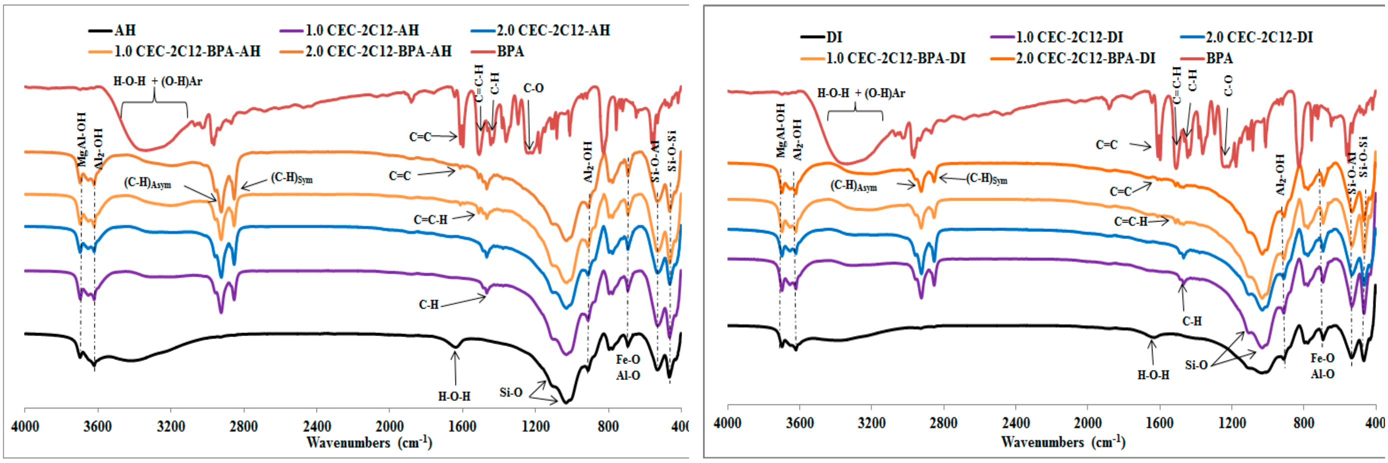

(**a**) (**b**)

Figure S4. IR spectra of (**a**) AH, BPA, $2C_{12}$-AH, $2C_{12}$-BPA-AH, (**b**) DI, BPA, $2C_{12}$-DI, $2C_{12}$-BPA-DI.
